# Supplementary material for: Mitochondrial respiration controls neoangiogenesis during wound healing and tumour growth
Source: Nat Commun. 2020 Jul 21;11:3653. doi: 10.1038/s41467-020-17472-2 (PMC7374592; doi:10.1038/s41467-020-17472-2)
Supplement: Supplementary file 1 — Supplementary Information [file 41467_2020_17472_MOESM1_ESM.pdf]

Mitochondrial respiration controls neoangiogenesis during wound healing and tumour growth

Schiffmann, Werthenbach et al.

Supplementary materials include:

Supplementary Figures 1 to 5

Supplementary Tables 1 and 2

Supplementary Figure 1

a

*Tie2Cre cox10^fl/wt^* x *cox10^fl/fl^*

genotype (*cox10 EC*)	+/+	+/-	-/-	total
obtained	49 (64.5%)	27 (35.5%)	0 (0%)	76 (100%)
expected	38 (50%)	19 (25%)	19 (25%)	76 (100%)

b

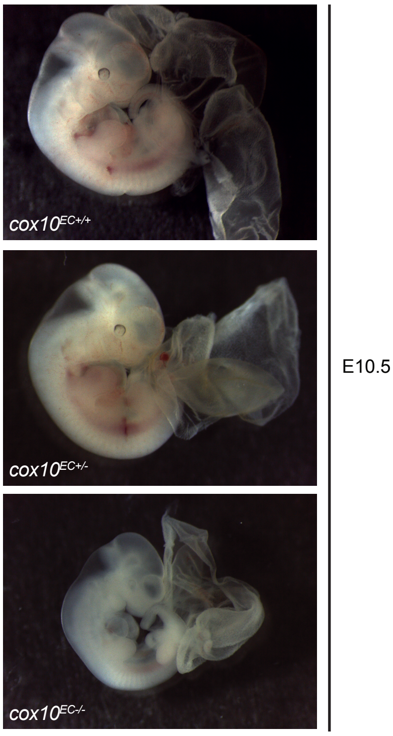

c

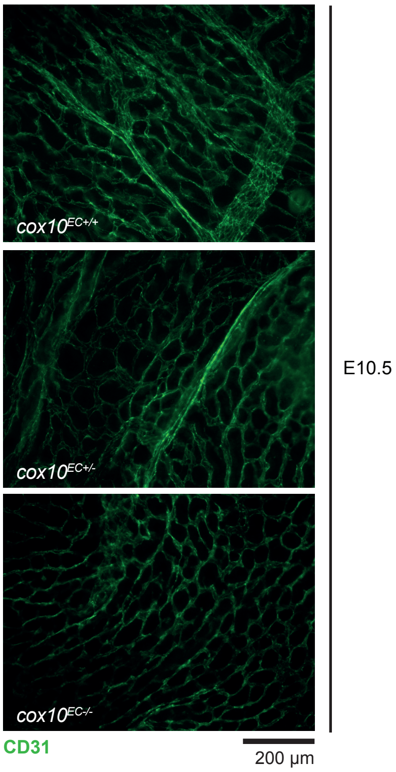

d

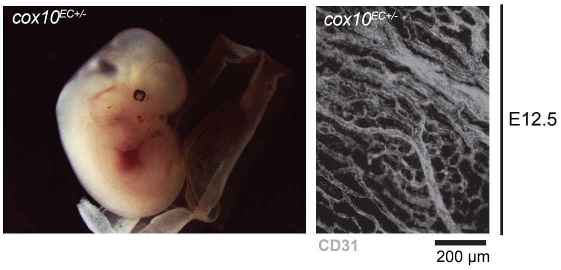

e

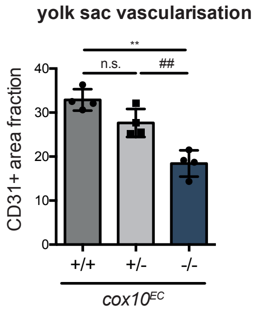

f

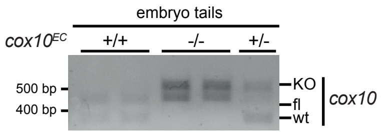

g

*LysMCre^Tg/wt^ cox10^fl/wt^* x *LysMCre^Tg/wt^ cox10^fl/wt^*

*LysMCre^Tg/Tg^ cox10^fl/wt^* x *LysMCre^Tg/Tg^ cox10^fl/wt^*

genotype (*cox10^MM^*)	+/+	+/-	-/-	total
obtained	28 (35%)	41 (51%)	11 (14%)	80 (100%)
expected	31 (39%)	33 (40%)	16 (21%)	80 (100%)

h

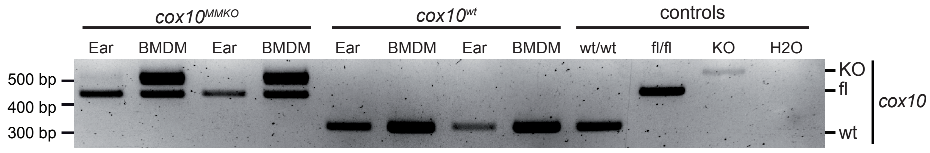

Supplementary Figure 1: Endothelial *cox10* is required for embryonic development

a, breeding scheme and table depicting expected and obtained (mendelian ratio %) progeny.

b, representative images of embryos with the respective genotypes at day E10.5. **c**, representative fluorescent images of E10.5 whole mount yolk sacs stained with anti-CD31 antibody. **d**, representative E12.5 heterozygous *cox10 EC*-deficient embryo (left panel) and the corresponding yolk sac whole mount stained for CD31. **e**, quantification of yolk sac vascularisation of E 12.5 embryos representing all 3 genotypes (compare Fig. 1 c). **f**, *cox10* PCR analysis of dissected *cox10* $^{EC+/+}$, *cox10*$^{EC+/-}$ and *cox10*$^{EC-/-}$ embryos. **g**, breeding scheme and table depicting expected and obtained (mendelian ratio %) progeny from crossing *LysMCre*$^{Tg/Tg}$ *cox10*$^{fl/wt}$ to *LysMCre*$^{Tg/Tg}$ *cox10*$^{fl/wt}$ or *LysMCre*$^{Tg/wt}$ *cox10*$^{fl/wt}$ to *LysMCre*$^{Tg/wt}$ *cox10*$^{fl/wt}$. **h**, PCR analysis of isolated bone marrow derived macrophages (BMDMs) and ear tags of either *cox10*$^{fl/fl}$ *LysMCre*$^{tg/tg}$ (*cox10*^{MMKO}) or *cox10*$^{wt/wt}$ *LysMCre*$^{tg/tg}$ (*cox10*^{wt}). PCR controls were derived from ear cuts (wt/wt and fl/fl) or cre treated MAFS isolated from *cox10*$^{fl/fl}$ mice (KO). Data in (**e**) are presented as mean ± SD. Individual data points in **e** represent analysed mice per genotype n=4. Exact p-values (two-way ANOVA, Bonferroni post-hoc): **: 0.0002, n.s.: 0.0898, ##: 0.0043.

Supplementary Figure 2

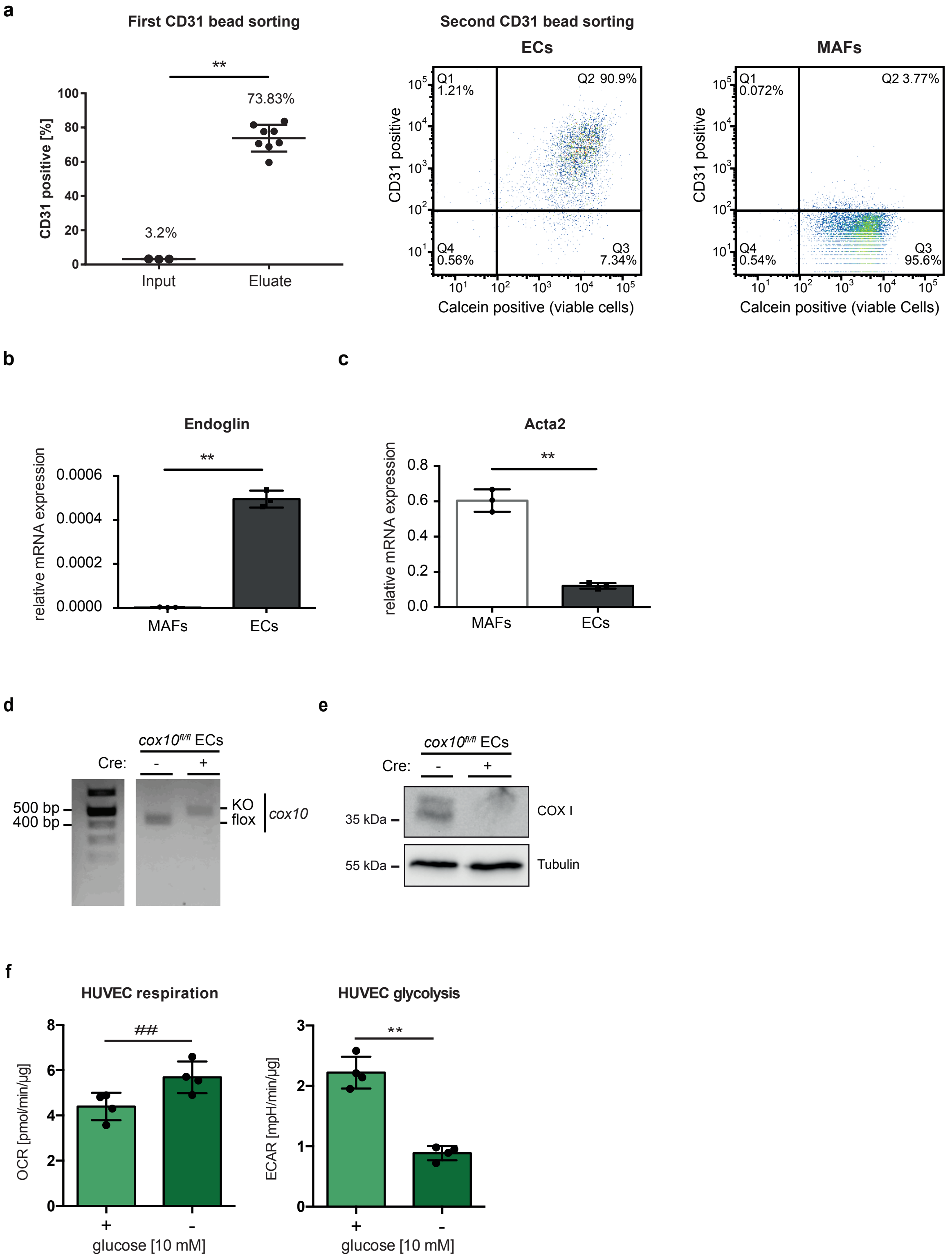

Supplementary Figure 2: Loss of endothelial *cox10* varies EC metabolic phenotype

a, fluorescent activated cell sorting (FACS) of lung cells before (input) and after (eluate) EC enrichment via CD31 bead sorting (First sorting (left panel), re-sorting (middle panel), negative control (murine adult fibroblasts, right panel)). Cells were labelled with CD31 antibody alone or together with calcein as a viability marker. **b, c,** quantitative PCR analysis showing relative mRNA levels of specific markers ((**b**) Endoglin, (**c**) Acta2) of murine fibroblasts vs. isolated primary murine ECs. **d**, PCR analysis of isolated untreated and Cre-treated $cox10^{fl/fl}$ ECs. **e**, Western blot of protein levels of COX subunit I of isolated untreated and Cre treated $cox10^{fl/fl}$ ECs. Tubulin served as loading control. **f,** OCR and ECAR of human umbilical vein endothelial cells (HUVECs) in the presence and absence of glucose. Data are presented as mean ± SD. Individual data points in (**a**), (**b**), (**c**), (**f**) represent technical replicates within a representative experiment. Sample size: (**a**): n=3 (Input), n=8 (eluate), (**b**): n=3 vs. n=3, (**c**): n=3 vs. n=3, (**f**): n=4 for each group. Exact p-values (unpaired students t-test, two tailed): (**a**): <0.0001, (**b**): <0.0001, (**c**): 0.0002. (**f**) ## 0.0079 ** 0.0007.

Supplementary Figure 3

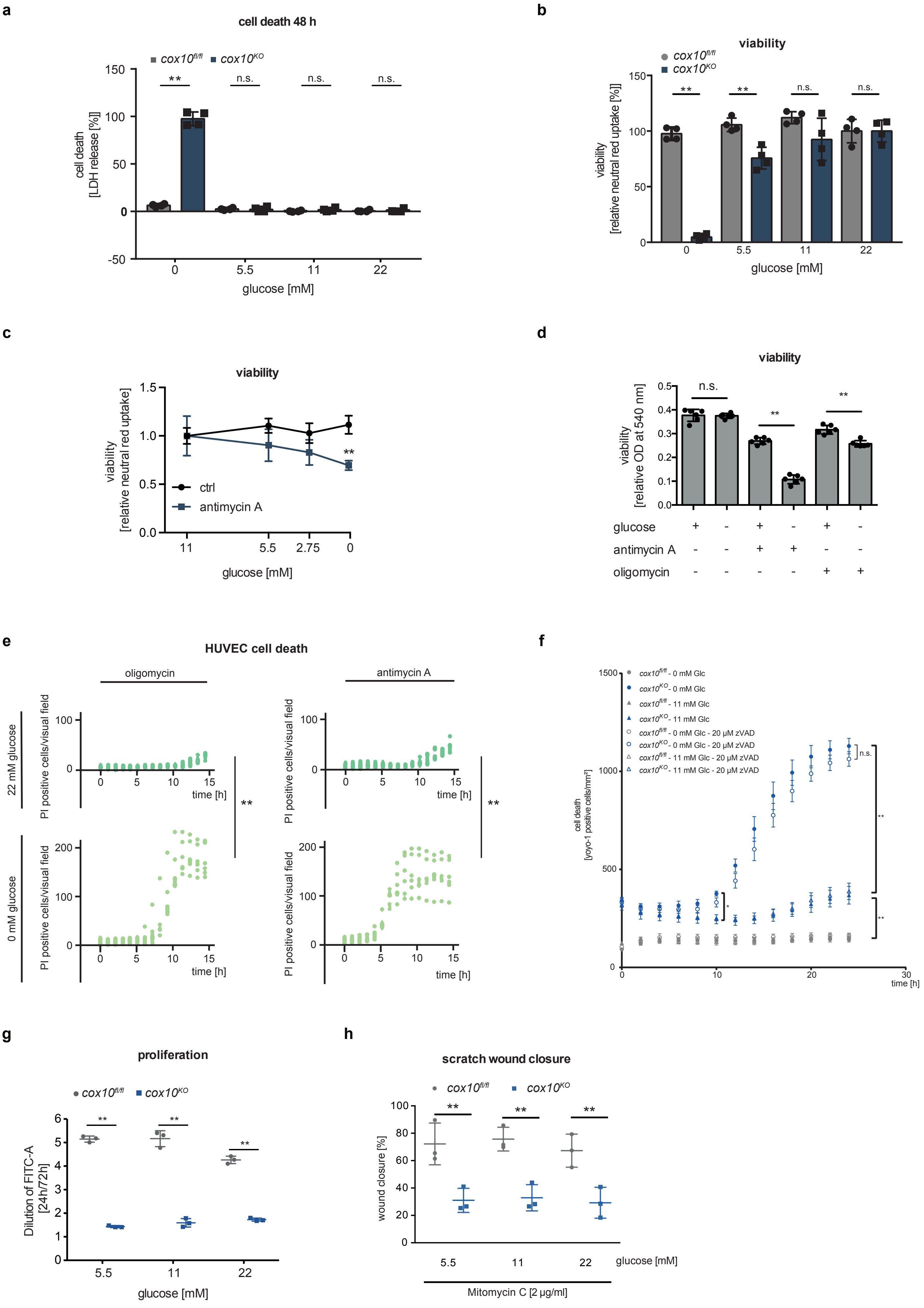

Supplementary Figure 3: *Cox10* is essential for EC function

a, cell death of *cox10*$^{fl/fl}$ ECs and *cox10*^{KO} ECs with increasing concentrations of glucose after 48 h and measured by LDH release. **b**, viability of *cox10*$^{fl/fl}$ ECs and *cox10*^{KO} ECs subjected to 0 mM, 5.5 mM, 11 mM and 22 mM glucose over 48 h and measured by neutral red uptake. **c,** viability of *cox10*$^{fl/fl}$ and *cox10*^{KO} ECs under decreasing concentrations of glucose left untreated (ctrl) or after treatment with antimycin A (10µm) measured by neutral red uptake. **d**, viability of primary murine wildtype ECs in 0 mM glucose vs. 22 mM glucose conditions in response to pharmacological targeting of the respiratory chain (oligomycin 2 µM, antimycin A 10 µM). **e**, kinetics of cell death (PI uptake) using a live imaging microscopic quantification of HUVECs exposed to oligomycin or antimycin A in the presence of 0 mM glucose vs. 22 mM glucose. **f**, live cell death analysis of *cox10*$^{fl/fl}$ and *cox10*^{KO} ECs treated with 0 mM and 11 mM glucose over 24h in the presence or absence of zVAD-fmk (20 µM) monitored with an incucyte system. **g**, proliferation of *cox10*$^{fl/fl}$ and *cox10*^{KO} ECs at indicated glucose concentrations over 72 h. **h**, quantification of scratch wound closure of monolayer cultured control *cox10*$^{fl/fl}$ and *cox10*^{KO} ECs (% of wound closure) at indicated glucose levels after 16 h in the presence of mitomycin C. Data are presented as mean ± SD. Sample sizes: (**a**) n=4, (**b**) n=4, (**c**) n=3 (**d**) n=5, (**e**) n=6, (**f**) n=3, (**g**) n=3, (**h**) n=3. Individual data points in (**a**), (**b**), (**c**), (**d**), (**e**), (**f**), (**g**) and (**h**) represent technical replicates within a representative experiment. Exact p-values (unpaired students t-test, two tailed): (**a**): <0.0001, n.s. (from left to right): 0.7851, 0.1657, 0.6949; **(b):** from left to right: <0.001, 0.0066, 0.2631, >0.9999; **(c)**: 0.0023; (**d**) ** < 0.0001; Two-way ANOVA followed by Bonferroni post-hoc analysis comparing mean PI values at t=15 h within treatment groups stratifying for glucose concentrations: **(e)** both ** <0.0001). P-values (two-way ANOVA followed by Bonferroni post-hoc test): (**f**): **<0.0001 *<0.0005, n.s. 0.0925; (**g**) **<0.0001. Multiple t-tests (two tailed) followed by Sidak-bonferroni method: (**h**): 5.5: 0.0155, 11: 0.0045, 22: 0.0162.

Supplementary Figure 4

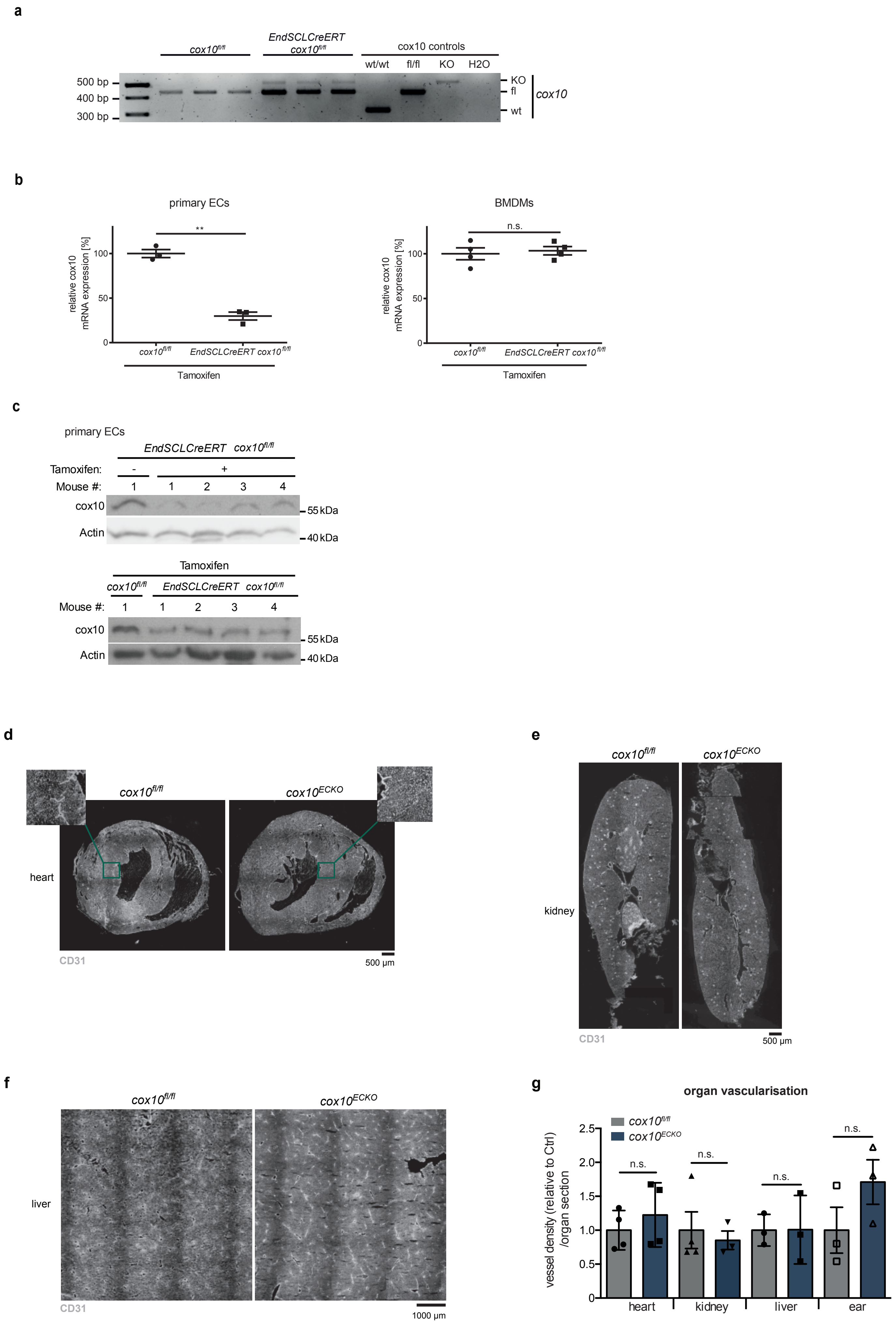

Supplementary Figure 4: *Cox10* is essential for EC function

a, PCR analysis of ear cuts from tamoxifen treated *cox10*$^{fl/fl}$ and *EndSCLCreERT cox10*$^{fl/fl}$ animals. **b**, *cox10* RT-PCR of freshly isolated primary lung endothelial cells and bone marrow derived macrophages from tamoxifen treated *cox10*$^{fl/fl}$ and *EndSCLCreERT cox10*$^{fl/fl}$ animals. **c**, COX10 Western blot analysis of freshly isolated primary lung endothelial cells from *cox10*$^{fl/fl}$ or *EndSCLCreERT cox10*$^{fl/fl}$ animals treated with tamoxifen or left untreated as indicated.

d, representative fluorescent images of CD31 stained hearts from *cox10*$^{fl/fl}$ control vs. *cox10*^{ECKO} mice. **e**, CD31 stained kidneys from *cox10*$^{fl/fl}$ control vs. *cox10*^{ECKO} mice. **f**, multiple image alignment of liver sections from *cox10*$^{fl/fl}$ control vs. *cox10*^{ECKO} mice stained for CD31. **g**, quantification of organ vascularisation in mice of indicated genotype. Data are presented as mean ± SD. Sample sizes:(**b**) n=3 vs n=3 (primary ECs) and n=4 vs n=4 (BMDMs). (**g**): n=4 (wt) vs. 4 (*cox10*^{ECKO}) (heart), n=4 vs. 3 (kidney), n=3 vs. 3 (liver, ear). Individual data points in (**g**) represent mean values of individual mice of the respective genotype. Exact p-values (unpaired students t-test, two-tailed): (**b**) **: 0.0004 n.s.: 0.6866 (**g**): 0.4485 (heart), 0.6794 (kidney), 0.9826 (liver), 0.2061 (ear).

Supplementary Figure 5

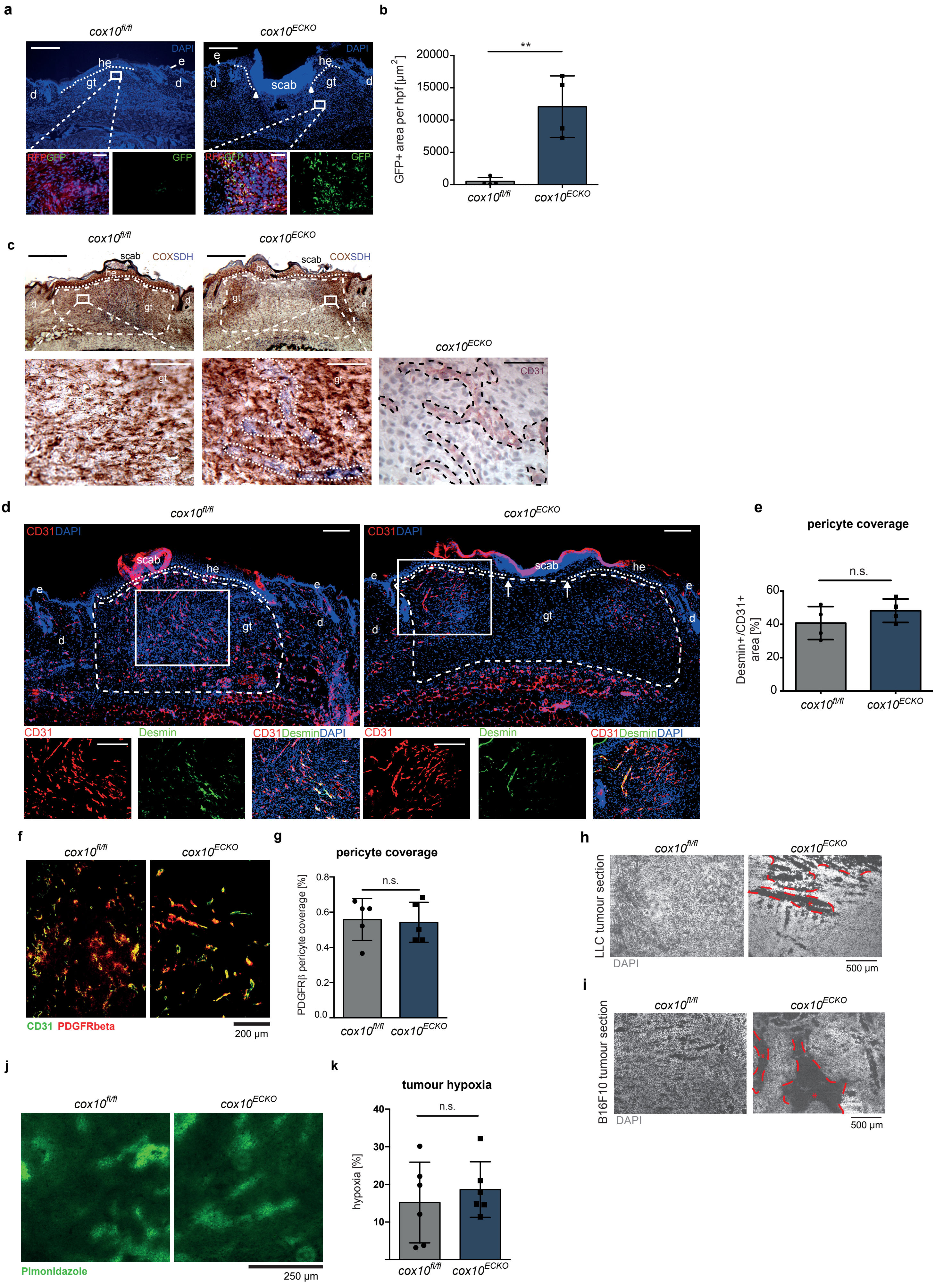

Supplementary Figure 5: EC OxPhos is required for neoangiogenesis in wound healing and tumour growth

a, Representative fluorescent wound sections and **b**, quantification of GFP+ area per hpf of mice with indicated genotypes, for data obtained in **a** and **b** mice were crossed to R26mTmG reporter mice (*cox10*$^{fl/fl}$ R26mTmG and *EndSCLCreERT cox10*$^{fl/fl}$ R26mTmG). Tamoxifen treatment in these mice lead to EC specific GFP expression only in *cox10*^{ECKO} mice; scale bar upper panel, 500 µm; scale bar lower panel, 50 µm. **c**, COX/SDH staining of mice with indicated genotypes (left panel) and CD31 immunostaining on a serial section in wound tissue from a *cox10*^{ECKO} mouse (right panel); scale bar upper panel, 500 µm; scale bar lower panel, 50 µm. d, dermis; e, epidermis; gt, granulation tissue; he, hyperproliferative epithelium. **d,** CD31 and Desmin immunostainings of wound sections at day 7 post injury from tamoxifen treated *cox10*$^{fl/fl}$ and *EndSCLCreERT cox10*$^{fl/fl}$ (*cox10*^{ECKO}) mice. Dotted line indicates the newly formed epithelium; dashed line indicates granulation tissue; arrows indicate the tips of the epithelial tongue; rectangles in the upper panel are highlighted in the lower panel; d: dermis, e: epidermis, gt: granulation tissue, he: hyperproliferative epithelium, scale bar: 200 µm. **e**, quantification of the ratio of Desmin+ area versus CD31+ area within the granulation tissue. **f**, representative images of CD31+ tumour vessels (green) and PDGFRbeta stained pericytes (red) and **g,** quantification thereof. **h/i**, representative fluorescent images of LLC (**h**) or B16F10 (**i**) tumour sections of *cox10*$^{fl/fl}$ control vs. *cox10*^{ECKO} mice stained for DAPI (asterisks marking necrotic areas). **j**, representative fluorescence images of pimonidazole stained LLC tumour tissue and **k**, quantification thereof. Data are presented as mean ± SD. Individual data points in (**b**), (**e**), (**g**), (**k**) represent mean values of individual mice of the respective genotype. Sample sizes: (**b**), (**e**): n=4 for both genotypes, (**g**) n=5 for both genotypes, (**k**): n=6 for both genotypes. Exact p-values, (unpaired students t-test, two tailed): (**b**): 0.0030**,** (**e**): 0.2665, (**g**): 0.8395, (**k**): 0.5316.

Supplementary Table 1: Primer list

Locus:	Primer name:	Sequence:
LysM Cre	Lys3	CCC AGA AAT GCC AGA TTA CG
LysM Cre	Lys1	CTT GGG CTG CCA GAA TTT CTC
LysM Cre	Lys2	TTA CAG TCG GCC AGG CTG AC
Tek Cre	Tek Cre 800	GCT GCC ACG ACC AAG TGA CAG CAA TG
Tek Cre	Tek Cre 1200	GTA GTT ATT CGG ATC ATC AGC TAC AC
mTmG	mTmG_wt_forw	CTC TGC TGC CTC CTG GCT TCT
mTmG	mTmG_wt_rev	CGA GGC GGA TCA CAA GCA ATA
mTmG	mTmG_tg_rev	TCA ATG GGC GGG GGT CGT T
EndSCL Cre	987	ATG TTT AGC TGG CCC AAA TG
EndSCL Cre	988	GCA ACG TGC TGG TTA TTG TG
EndSCL Cre	989	AGC ATG CTC TTT TCC AGC AT
EndSCL Cre	990	CTC AGG CTG GCC TAA AAC TG
Actin	FW	CTG AGA GGG AAA TCG TGC GT
Actin	RV	AAC CGC TCG TTG CCA ATA GT
Cox10	FW	AAG AAC AGG CCT CTG GTT CG
Cox10	RV	GCT CCA ACC CAG GTA TTG GT
Endoglin	FW	CCC TCT GCC CAT TAC CCT G
Endoglin	RV	GTA AAC GTC ACC TCA CCC CTT
Acta2	FW	GTC CCA GAC ATC AGG GAG TAA
Acta2	RV	TCG GAT ACT TCA GCG TCA GGA

Supplementary Table 2: Antibody list

Reference	Antibody	Company
cat. No.: 102515	Alexa Fluor® 647 anti-mouse CD31 Antibody	Biolegend
cat. No.: 102406	FITC anti-mouse CD31 Antibody	Biolegend
cat. No.: ab119341	Anti-CD31 antibody [2H8]	Abcam
cat. No.: ab109025	Anti-COX1 / Cyclooxygenase 1 antibody [EPR5866]	Abcam
cat. No.: T9026	Anti alpha-Tubulin	Sigma-Aldrich
cat. No.: 10611-2-AP	Rb pAb COX10	Proteintech
cat. No.: sc-47778 HRP	β-Actin Antikörper (C4) HRP	Santa Cruz
cat. No.: 14-1402-82	PDGFR CD140b mouse	eBiosciences
cat. No.: ab173004	Goat Anti-Armenian hamster IgG H&L (Alexa Fluor® 647)	Abcam
cat. No.: A-21244	Goat anti-Rabbit IgG (H+L) Cross-Adsorbed Secondary Antibody, Alexa Fluor 647	ThermoFisher Scientific
cat. No.: #7074	Anti-rabbit IgG, HRP-linked Antibody	Cell Signaling
