# Supplementary material for: Mitochondrial respiration controls neoangiogenesis during wound healing and tumour growth
Source: Nat Commun. 2020 Jul 21;11:3653. doi: 10.1038/s41467-020-17472-2 (PMC7374592; doi:10.1038/s41467-020-17472-2)
Supplement: Supplementary file 3 — Description of Additional Supplementary Files [file 41467_2020_17472_MOESM3_ESM.docx]

Description of Additional Supplementary Files

File Name: Supplementary Movie 1
Description: Video of 3D projection depicting the vasculature (visualised by CD31 staining) of wholemount cox10^EC-/-^ embryos at E12.5 (5x magnification).

File Name: Supplementary Movie 2
Description: Video of z-plane compilation depicting the vasculature (visualised by CD31 staining) of wholemount cox10^EC-/-^ embryos at E12.5 (5x magnification).

File Name: Supplementary Movie 3
Description: Video of z-plane compilation depicting the vasculature (visualised by CD31 staining) of wholemount cox10^EC-/-^ embryos at E12.5 (10x magnification).

File Name: Supplementary Movie 4
Description: Video of 3D projection depicting the vasculature (visualised by CD31 staining) of wholemount cox10^EC+/+^ embryos at E12.5 (5x magnification).

File Name: Supplementary Movie 5
Description: Video of z-plane compilation depicting the vasculature (visualised by CD31 staining) of wholemount cox10^EC+/+^ embryos at E12.5 (5x magnification).

File Name: Supplementary Movie 6
Description: Video of z-plane compilation depicting the vasculature (visualised by CD31 staining) of wholemount cox10^EC+/+^ embryos at E12.5 (10x magnification).
