# Supplementary material for: Mitochondrial respiration controls neoangiogenesis during wound healing and tumour growth
Source: Nat Commun. 2020 Jul 21;11:3653. doi: 10.1038/s41467-020-17472-2 (PMC7374592; doi:10.1038/s41467-020-17472-2)
Supplement: Supplementary file 10 — Reporting Summary [file 41467_2020_17472_MOESM10_ESM.pdf]

natureresearch

Corresponding author(s): Hamid Kashkar

Last updated by author(s): 13/05/2020

Reporting Summary

Nature Research wishes to improve the reproducibility of the work that we publish. This form provides structure for consistency and transparency in reporting. For further information on Nature Research policies, seeAuthors & Referees and theEditorial Policy Checklist .

Statistics

For all statistical analyses, confirm that the following items are present in the figure legend, table legend, main text, or Methods section.

n/a	Confirmed	
☐	☒	The exact sample size (*n*) for each experimental group/condition, given as a discrete number and unit of measurement
☐	☒	A statement on whether measurements were taken from distinct samples or whether the same sample was measured repeatedly
☐	☒	The statistical test(s) used AND whether they are one- or two-sided<br>*Only common tests should be described solely by name; describe more complex techniques in the Methods section.*
☒	☐	A description of all covariates tested
☐	☒	A description of any assumptions or corrections, such as tests of normality and adjustment for multiple comparisons
☐	☒	A full description of the statistical parameters including central tendency (e.g. means) or other basic estimates (e.g. regression coefficient) AND variation (e.g. standard deviation) or associated estimates of uncertainty (e.g. confidence intervals)
☐	☒	For null hypothesis testing, the test statistic (e.g. *F*, *t*, *r*) with confidence intervals, effect sizes, degrees of freedom and *P* value noted<br>*Give P values as exact values whenever suitable.*
☒	☐	For Bayesian analysis, information on the choice of priors and Markov chain Monte Carlo settings
☒	☐	For hierarchical and complex designs, identification of the appropriate level for tests and full reporting of outcomes
☒	☐	Estimates of effect sizes (e.g. Cohen's *d*, Pearson's *r*), indicating how they were calculated

Our web collection on statistics for biologists contains articles on many of the points above.

Software and code

Policy information about availability of computer code

Data collection	CellR Imaging Software, Diskus software, Seahorse Wave Desktop Software, Leica LAS X Software, Microsoft Excel, ImageJ, LinRegPCR, Adobe Illustrator
Data analysis	Graphpad Prism V6 and R version 3.5.3 (2019-03-11) (http://www.R-project.org) was used to analyse data in this study.

For manuscripts utilizing custom algorithms or software that are central to the research but not yet described in published literature, software must be made available to editors/reviewers. We strongly encourage code deposition in a community repository (e.g. GitHub). See the Nature Research guidelines for submitting code & software for further information.

Data

Policy information about availability of data

All manuscripts must include a data availability statement. This statement should provide the following information, where applicable:
- Accession codes, unique identifiers, or web links for publicly available datasets
- A list of figures that have associated raw data
- A description of any restrictions on data availability

The source data underlying the main figures and extended data figures are provided as a source data file. Other datasets generated and analysed for this work are accessible upon request.

Field-specific reporting

Please select the one below that is the best fit for your research. If you are not sure, read the appropriate sections before making your selection.

Life sciences study design

All studies must disclose on these points even when the disclosure is negative.

Sample size	For in vitro experiments, sample sizes were based on previous publications without prior power analysis.<br>For mouse experiments sample sizes were determined by prior determined power analysis as requested by the german aninal law.
Data exclusions	No data were excluded
Replication	All data presented in the study were reliably reproduced. In vitro experiments were repeated at least two times. Data involving animals depict pooled data of at least three independent experiments.
Randomization	No randomization was performed.
Blinding	For embryology experiments embryos were imaged blinded before genotyping. Tumor measurments were performed blinded. Image aquiring during fluorescence microscopy and analysis with imageJ was performed by an investigator blinded to the genotype. Cell culture experiments were not blinded due to practical and personell reasons.

Reporting for specific materials, systems and methods

We require information from authors about some types of materials, experimental systems and methods used in many studies. Here, indicate whether each material, system or method listed is relevant to your study. If you are not sure if a list item applies to your research, read the appropriate section before selecting a response.

Materials & experimental systems

n/a	Involved in the study	
☐	☒	Antibodies
☐	☒	Eukaryotic cell lines
☒	☐	Palaeontology
☐	☒	Animals and other organisms
☒	☐	Human research participants
☒	☐	Clinical data

Methods

n/a	Involved in the study	
☒	☐	ChIP-seq
☐	☒	Flow cytometry
☒	☐	MRI-based neuroimaging

Antibodies

Antibodies used	Alexa Fluor® 647 anti-mouse CD31 Antibody, Biolegend, cat. No.: 102516, clone MEC 13.3<br>FITC anti-mouse CD31 Antibody, Biolegend, cat. No.: 102406, clone 390<br>Anti-CD31 antibody, Abcam, cat. No.: ab119341, clone 2H8<br>Anti-COX1 / Cyclooxygenase 1 antibody, Abcam, cat. No.: ab109025, clone EPR5866<br>Anti alpha-Tubulin, Sigma-Aldrich, cat. No.: T9026, clone: DM1A<br>Rb pAb COX10, Proteintech, cat. No.: 10611-2-AP<br>β-Actin Antikörper HRP, Santa Cruz, cat. No.: sc-47778 HRP, clone C4<br>PDGFR CD140b mouse, eBiosciences, cat. No.: 14-1402-82, clone APB5<br>Goat Anti-Armenian hamster IgG H&L (Alexa Fluor® 647), Abcam, cat. No.: ab173004<br>Goat anti-Rabbit IgG (H+L) Cross-Adsorbed Secondary Antibody, Alexa Fluor 647, ThermoFisher Scientific, cat. No.: A-21244<br>Anti-rabbit IgG, HRP-linked Antibody, Cell Signaling, cat. No.: #7074
Validation	All antibodies used in this study are commercially available and were validated by the supplier. Appropriate controls were used within experiments where needed. Please refer to the manufacturers' websites for additional information regarding validation protocols and resources.

Eukaryotic cell lines

Policy information about cell lines

Cell line source(s)	LLC, B16F10: both from ATCC. HUVECs were purchased from PromoCell.
Authentication	None of the cell lines were re-authenticated, as these cells are easily to distinguish morphologically in culture.
Mycoplasma contamination	Cell lines were tested negative for Mycoplasma before every experiment
Commonly misidentified lines<br>(See ICLAC register)	No commonly misidentified cell lines were used in this study.

Animals and other organisms

Policy information about studies involving animals; ARRIVE guidelines recommended for reporting animal research

Laboratory animals	EndSCLCreERT cox10 fl/fl and Tie2Cre cox10 fl/wt mice were on a mixed C57BL/6N and C57BL/6J background. EndSCLCreERT cox10 fl/fl R26mTmg mice were on a mixed C57BL/6N , C57BL/6J and CD1 background. Animals were housed in the animal care facility of the University of Cologne under standard pathogen-free conditions with a 12 h light/dark schedule and provided with food and water ad libitum, the temperature was between 20-24°C and relative humidity between 45-65 rH. Mice were between 8 and 16 weeks old, male and female mice were used for experiments.
Wild animals	did not involve wild animals
Field-collected samples	did not involve field-collected samples
Ethics oversight	All mouse studies were performed with approval by local government authorities (LANUV, NRW, Germany) in accordance with the German animal protection law.

Note that full information on the approval of the study protocol must also be provided in the manuscript.

Flow Cytometry

Plots

Confirm that:

- [x] The axis labels state the marker and fluorochrome used (e.g. CD4-FITC).
- [x] The axis scales are clearly visible. Include numbers along axes only for bottom left plot of group (a 'group' is an analysis of identical markers).
- [x] All plots are contour plots with outliers or pseudocolor plots.
- [x] A numerical value for number of cells or percentage (with statistics) is provided.

Methodology

Sample preparation	Proliferation of ECs was assessed using the CellTrace™ CFSE Cell Proliferation Kit, for flow cytometry (Thermo Fisher Scientific, USA) according to the manufacturer's instructions. 40.000 cells were seeded in 12-wells and stained with CellTrace™ CFSE in triplicate. The fluorescent staining was analyzed by fluorescence activated cell sorting (FACS) after 24h and 72h.
Instrument	BD FacsCanto (BD BioSciences, USA)
Software	BD FACSDiva (V. 5.0.3) software for aquisation of FACS data and FlowJo10 for data analysis.
Cell population abundance	10,000 cells were gated per replicate.
Gating strategy	Viable cells were defined and gated between approximately 40k and 250k FSC and approximately 5k and 260k SSC.

- [x] Tick this box to confirm that a figure exemplifying the gating strategy is provided in the Supplementary Information.
